# Spontaneous Hyperinflation of Intragastric Balloon Presenting as Acute Pancreatitis

**DOI:** 10.1002/deo2.70414

**Published:** 2026-08-26

**Authors:** Ong Yan Ling, Rajesh R, Asokkumar Ravishankar

**Affiliations:** ^1^ Department of Gastroenterology and Hepatology Singapore General Hospital Singapore Singapore; ^2^ Duke–NUS Graduate Medical School Singapore Singapore

**Keywords:** acute pancreatitis, balloon hyperinflation, *Candida*, endoscopic bariatric therapy, intragastric balloon

## Abstract

Obesity is a metabolic disorder with considerable health implications affecting populations worldwide. Intragastric balloon (IGB) placement has gained popularity as a minimally invasive and reversible management option for obesity. Although generally safe, IGBs can sometimes be associated with serious adverse outcomes. We present a case of IGB hyperinflation due to *Candida* infection, resulting in local compression of the pancreas and causing acute pancreatitis. Urgent endoscopic removal of the balloon led to complete resolution of symptoms. Spontaneous hyperinflation of an IGB due to fungal overgrowth can lead to extrinsic pancreatic compression. Early recognition and timely balloon removal are key to the management of IGB‐related acute pancreatitis.

## 1 Introduction

Obesity is a global metabolic disease associated with substantial morbidity, including increased risk of cardiometabolic conditions, musculoskeletal disorders, and psychosocial burden, often translating into significant socioeconomic impact. Management strategies span lifestyle modification, pharmacotherapy, and procedural interventions, including endoscopic and surgical therapies. Intragastric balloon (IGB) placement is a well‐established, minimally invasive endoscopic bariatric therapy that achieves clinically meaningful weight loss at 6 and 12 months [1].

Although IGBs are generally safe, adverse events can occur. Transient symptoms such as nausea, vomiting, and abdominal discomfort are reported in up to 20% of patients [2]. Serious complications are uncommon (<1%) but may include gastric ulceration, perforation, and intestinal obstruction, with reported mortality as low as 0.05%. Acute pancreatitis represents a rare but recognized complication following IGB placement. However, its true incidence remains uncertain as existing literature consists predominantly of case reports and small retrospective series [3].

We report a rare case of spontaneous IGB hyperinflation secondary to polymicrobial Candida infection, resulting in compressive pancreatitis, which was successfully managed with prompt endoscopic balloon removal.

## 2 Case Report

A 24‐year‐old woman with a background of major depressive disorder and non‐alcoholic fatty liver disease presented for weight management (body mass index [BMI] 28 kg/m^2^). Having been unsuccessful in achieving weight loss through dietary and lifestyle modification, she underwent endoscopic placement of a fluid‐filled IGB (Orbera, Boston Scientific, USA). The balloon was filled with 650 mL of sterile saline mixed with methylene blue. Baseline endoscopy was unremarkable. She was discharged on proton pump inhibitors and antiemetics.

The patient initially tolerated the balloon well and had lost 12 kg since insertion. However, four months post‐placement, she presented with acute‐onset vomiting, abdominal pain, and progressive abdominal distension. Vital signs were stable (temperature 36.6°C, heart rate 66 bpm, blood pressure 109/66 mmHg). On examination, there was a visible and palpable mass in the left upper quadrant. Laboratory investigations demonstrated a mild metabolic alkalosis (pH 7.44, bicarbonate 21.7 mmol/L), elevated serum amylase 216 U/L and lipase 155 U/L, with normal liver function tests.

**FIGURE 1:**
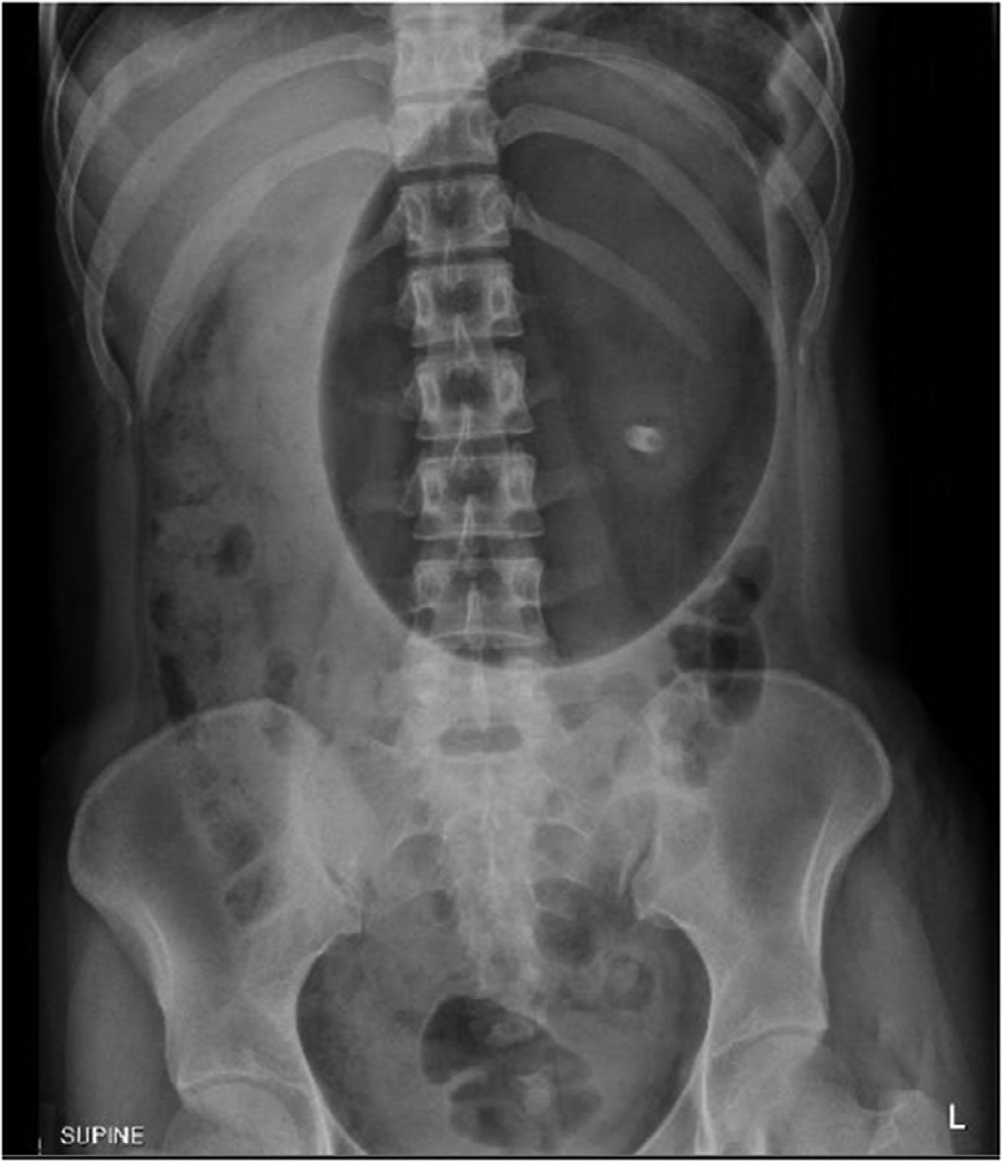
Abdominal radiograph (supine) demonstrating a grossly distended intragastric balloon with an air‐fluid level, without evidence of intestinal obstruction or free perforation.

Abdominal radiographs revealed a markedly enlarged balloon with a prominent air–fluid level. The estimated balloon volume was approximately 1437 mL, more than double the initial fill volume (Figure 1). All other etiologies of pancreatitis were excluded. A computed tomography scan was performed, which showed a distended balloon exerting pressure on the pancreas and features of pancreatitis.

**FIGURE 2:**
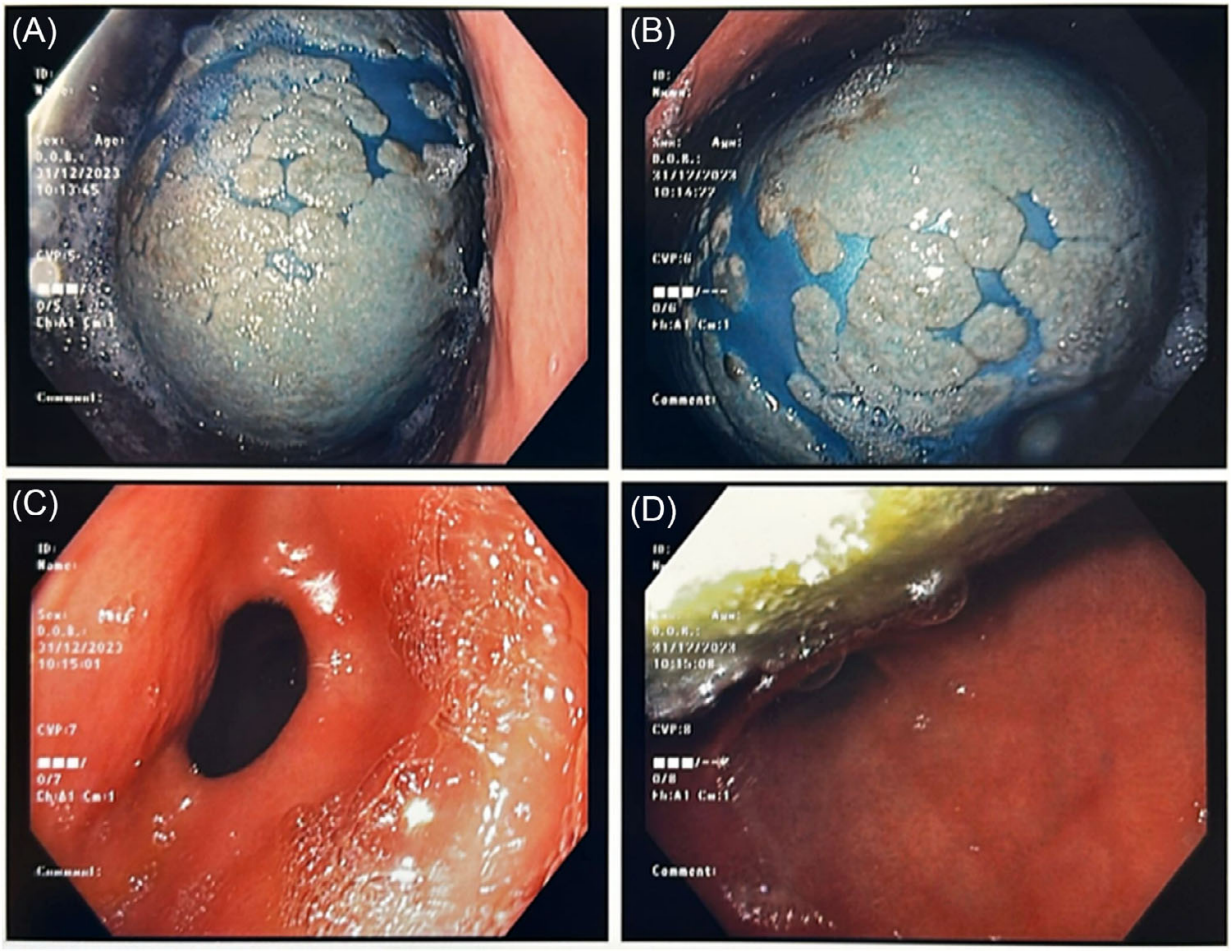
Endoscopic images at the time of balloon removal. (A, B) Hyperinflated intragastric balloon (IGB) coated with whitish fungal elements and blue dye, consistent with fungal biofilm. (C) Pyloric opening visualized following balloon deflation. (D) Balloon surface with adherent fungal material prior to extraction.

Urgent endoscopy confirmed a hyperinflated balloon with a large air–fluid interface coated with fungal elements (Figure 2). There were no overt signs of mucosal ischemia. The balloon was punctured, aspirated, and removed endoscopically without complication. Fluid aspirated from the balloon was sent for microbiological analysis.

Cultures grew *Candida tropicalis*, *Nakaseomyces glabrata*, and *Lactobacillus fermentum*. The patient experienced immediate symptomatic relief following balloon removal and recovered without the need for antifungal therapy.

At 6‐month follow‐up, she was well, with a BMI of 20.6 kg/m^2^, reflecting sustained weight loss following the procedure.

## 3 Discussion

IGB therapy involves endoscopic placement of a saline‐filled device (typically 400–700 mL) that promotes weight loss by inducing early satiety, delaying gastric emptying, and modulating appetite‐related hormones such as ghrelin [4]. It is used either as a bridge to bariatric surgery or as a primary intervention in patients unsuitable for more invasive procedures. Clinical studies have demonstrated meaningful weight loss at 6 months, with at least 10% total body weight loss achieved in a substantial proportion of patients [5].

Serious complications with IGBs are infrequent. Acute pancreatitis is an uncommon but recognized complication of IGB therapy. Proposed mechanisms include: (i) ampullary obstruction due to catheter migration into the duodenum (Spatz adjustable IGB); (ii) extrinsic compression of the pancreas from mass effect of the balloon [6]; and (iii) spontaneous balloon hyperinflation, as seen in this case, leading to progressive pancreatic compression. The latter mechanism is increasingly reported and is thought to be driven by microbial gas production within the balloon [7].

Under normal conditions, the stomach contains relatively low microbial counts, predominantly Gram‐positive organisms such as Streptococcus and Lactobacillus, with small amounts of *Candida* [8]. However, delayed gastric emptying induced by the balloon, together with proton pump inhibitor use, creates a favorable environment for fungal overgrowth and biofilm formation [9]. Microbial contamination may occur during insertion via oropharyngeal flora or through transmembrane permeation over time [7].

Candida species readily adhere to silicone surfaces and form biofilms, enhancing persistence and resistance to host defenses. Their transition to filamentous forms further supports colonization. Gas production by these organisms within the balloon is the most plausible driver of progressive hyperinflation and subsequent mass effect [10].

This case highlights several clinically relevant points. First, the polymicrobial culture (*C. tropicalis* and *N. glabrata*) aligns with prior reports suggesting synergistic biofilm formation in IGB‐related hyperinflation. Second, rapid resolution of pancreatitis following balloon removal underscores that early endoscopic intervention is definitive. Finally, sustained weight loss despite early removal reinforces the therapeutic value of IGBs, even when complications occur.

There is currently no standardized strategy to prevent IGB‐associated microbial overgrowth. Proposed approaches include antifungal prophylaxis, the use of bacteriostatic additives such as methylene blue, and selective surveillance in high‐risk patients (e.g., those with prolonged PPI use), although supporting evidence remains limited. Clinicians should maintain a high index of suspicion for balloon hyperinflation in patients presenting with new‐onset abdominal pain and distension after IGB placement, as timely recognition and intervention are critical.

Spontaneous hyperinflation should be considered in any patient with an IGB presenting with new‐onset abdominal pain and distension after the initial adaptation period. Early imaging and prompt endoscopic intervention are critical. This case supports the role of Candida‐associated gas formation as a key mechanism and highlights the importance of vigilance for this under‐recognized complication.

## Author Contributions

**Ong Yan Ling**: gathered clinical data and wrote the manuscript. **Rajesh R**: conceived the case report and wrote the manuscript. **Asokkumar Ravishankar**: provided overall supervision and contributed to the writing of the manuscript.

## Funding

The authors have nothing to report.

## Consent

Written informed consent was obtained from the patient for publication of this case report and accompanying images.

## Conflicts of Interest

The authors declare no conflicts of interest.
